# Assessing the contribution of mobility in the European Union to rubber expansion

**DOI:** 10.1007/s13280-021-01579-x

**Published:** 2021-06-12

**Authors:** Perrine C. S. J. Laroche, Catharina J. E. Schulp, Thomas Kastner, Peter H. Verburg

**Affiliations:** 1 Institute for Environmental Studies, Vrije Universiteit Amsterdam, De Boelelaan 1087, 1081 HV Amsterdam, The Netherlands; 2 Senckenberg Biodiversity and Climate Research Centre (SBiK-F), Senckenberganlage 25, 60325 Frankfurt-am-Main, Germany; 3 Swiss Federal Research Institute for Forest, Snow and Landscape Research WSL, Birmensdorf, Switzerland

**Keywords:** Carbon emission, Ecological impact, European Union, Natural rubber, Telecoupling, Transport

## Abstract

Nearly three-quarters of global natural rubber production is used to produce tyres, supporting mobility around the globe. The projected increase in mobility could contribute to further expansion of rubber plantations and impact tropical ecosystems. We quantified the use of natural rubber in tyres in the European Union (EU), the corresponding land footprint, and explored drivers of tyre use using country-specific transport statistics and trade registers of rubber goods. Five percent of the world’s natural rubber is consumed in tyres used in the EU, using up to a quarter of the area under rubber plantations in some producing countries. Car use is responsible for 58% of this consumption, due to car-dependent lifestyles that are associated with economic prosperity and spatial planning paradigms. While the EU’s transport policy focuses on reducing dependence on fossil-fuels, cross-cutting policies are needed to address car-dependency and reduce the EU’s land footprint in tropical landscapes without compromising progress towards decarbonisation.

**Supplementary Information:**

The online version contains supplementary material available at 10.1007/s13280-021-01579-x.

## Introduction

Over the past decade, demand for natural rubber has grown steadily by five percent every year (ETRMA 2019a) leading to the rapid expansion of rubber plantations in tropical countries (FAOSTAT 2019). High rubber prices on international markets have triggered many governments to promote rubber production as a way to fight rural poverty (Ahrends et al. 2015). Numerous smallholders have adopted rubber production and large rubber plantations have been established by agribusinesses (Warren-Thomas et al. 2015). While the primary hotspot for rubber expansion is Southeast Asia (Dove 2018), similar trends are being observed in other parts of Asia (Debbarma and Purkayasthaî 2019) and in Africa (Feintrenie 2014), and future expansion is expected in South America (Heer et al. 2015).

Rubber plantations have been associated with deforestation since colonial times (Lang 2001). More recently, rubber expansion has driven deforestation in China (Qiu 2009) and Southeast Asia (Reddy et al. 2016; Hurni and Fox 2018). In Sub-Saharan Africa, forested land has been converted into subsistence cropland near newly established rubber estates in order to meet the needs of local plantation workers (Feintrenie 2014). In many cases, rubber plantations have replaced forested land that had been degraded by years of shifting cultivation (Hurni and Fox 2018; Neyret et al. 2020). Due to its suitability to sloping terrain, rubber is additionally one of the few boom crops expanding into upland areas, threatening relatively untouched ecosystems such as the mountainous regions of Asia (Fox et al. 2014) or the central region of Côte d’Ivoire (Ouattara 2019).

Land use change associated with increasing rubber cultivation has ecological consequences, such as biodiversity loss and erosion. High erosion rates from intense rainfall were reported in upland rubber plantations (Ahrends et al. 2015) because soil quality and understory structure are poor compared to forests. Additionally, lower organic matter input (Van Straaten et al. 2015) and high fertilizer inflows (Berkelmann et al. 2020) within monoculture rubber plantations threaten multitrophic abundance and diversity (Barnes et al. 2014; Singh et al. 2019). Rubber plantations can support a diversity and abundance of some common animal species, but species of high conservation value prevail in natural forests in much larger numbers (Paoletti et al. 2018). Ecological disturbances are not as severe in rubber tree-based agroforestry systems, due to additional supply of organic matter and habitats (Liu et al. 2018; Warren-Thomas et al. 2020). Yet, currently agroforestry systems account for only half of the cultivated area in Indonesia and approximately 5% in Thailand (Penot et al. 2017).

Past trends have shown that the price of natural rubber is highly volatile in the long term (Grogan et al. 2019) due to the structure of the production sector, the seven-year ripening period, and the vulnerability of rubber trees to climatic events and pest outbreak (Meyer 2019; Millard 2019). Yet, prospects for rubber profits, and more recently carbon sink plantation plans and programmes (e.g. AFR100, the Bonn Challenge) (WRM 2020), are driving the global rubber boom, attracting smallholders (who supply 80% of world production), national governments, and investors into rubber production (Fox et al. 2014; Debbarma and Purkayasthaî 2019).

The automotive industry is the biggest rubber user globally, with over 70% of global natural rubber production being used for tyres (Millard 2019). Trend-based scenarios expect a tripling of freight transport and a doubling of passenger flows by 2050 (ITF 2019). Private motor vehicle use in cities is expected to decrease as shared mobility increases, but nonurban private transport is expected to increase as rising per capita income in many countries is likely to enable growth in car ownership. Additionally, increasing leisure time and tourism opportunities might increase demand for transport (ITF 2019). Transport growth projections for the European Union (EU) are very modest when compared to other world regions (ITF 2019), as current mobility levels are already relatively high by global standards. Nevertheless, mobility patterns are heterogeneous in the EU, as demonstrated by strong variations in car ownership (Focas and Christidis 2017) and mobility cultures (Haustein and Nielsen 2016).

The EU’s transport policy currently focuses exclusively on reducing greenhouse gas emissions from the sector to a level 60% lower than in 1990 by 2050 without curbing projected mobility growth (European Commission 2011). However, aiming for ever higher levels of mobility in the EU might lead to an increase in global rubber production, with repercussions to ecosystem functioning in the tropics. Therefore, the objective of this study is to assess the current contribution of mobility patterns in the EU to global natural rubber use and production. We estimate the annual use of natural rubber per vehicle category and spatially allocate its related land footprint. We then assess links to mobility patterns in the EU by comparing the rubber efficiency of overland vehicles and the correlations between per capita consumption of natural rubber in car tyres, and car use for everyday trips and long-distance travel for business or leisure.

## Materials and methods

**Fig. 1:**
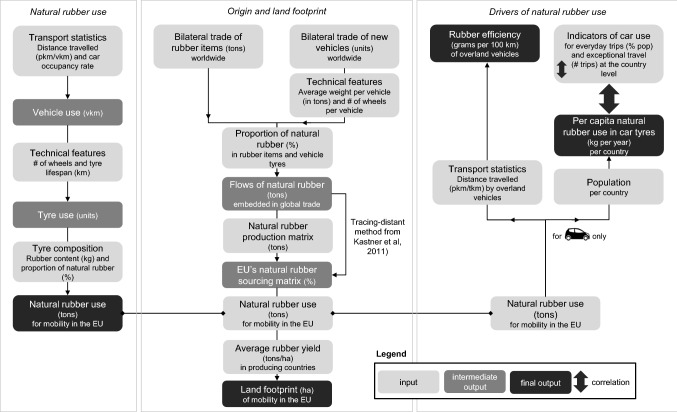
Overview of the methodology

We quantified the annual use of natural rubber for mobility in the EU based on transport statistics, and used bilateral trade records and production data to estimate and allocate the land footprint to countries where rubber is grown. We estimated the rubber efficiency of overland vehicles, i.e. the amount of natural rubber used to move a person or a metric ton of goods over a distance of 100 km, and calculated the per capita use of natural rubber in car tyres at the country level to assess correlations with car use for different purposes. The three components of our methodology are depicted in Fig. 1 and elaborated upon in detail in the following sections.

### Natural rubber use in the European Union

To assess the quantity of natural rubber needed to support mobility in the EU, we translated country-specific transport statistics for a wide range of vehicles with tyres (including aircrafts) into annual use of vehicles, tyres, and natural rubber, using a number of conversion factors (Fig. 1). We analysed the sensitivity of our results to variations in certain parameters for which substantially different values were found (see Fig. S2). Minimum impacts were calculated by applying the minimum number of tyres per vehicle, lowest natural rubber content, and highest tyre lifespan, while maximum impacts were obtained with the maximum number of tyres, highest natural rubber content, and lowest tyre lifespan.

#### Total vehicle use (vkm)

Tyre use depends on the total aggregated distance travelled annually by different categories of road vehicles, commonly labelled “vkm” in transport statistics. The latest vkm available for motorcycles and light vans are provided by TRACCS (2013) for 2010. For medium and heavy trucks, the latest vkm is reported by Eurostat (2020a, b) for 2017, broken down into 17 sub-categories according to the number of axles on front and towed vehicles. Only for Belgium, Hungary, Italy, Malta, and the Netherlands, we lacked data, so we used the vkm provided by TRACCS (2013) for 2010, matching the truck categories according to the maximum authorized weight (Gov.UK 2013) (Table S1). For cars and buses, DG MOVE (2018) provides the total aggregated distance travelled in 2017 by all passengers, commonly labelled “pkm” in transport statistics. We converted the pkm into vkm, following Eq. (1), using country-specific occupancy rates, i.e. the average number of people traveling in one vehicle per trip, from Fiorello and Zani (2018) for cars and from TRACCS (2013) for 2010 for buses.

1 $$  {\text{vkm}}\;\left({{\text{e.g.}}\;{\text{cars}}} \right) = \frac{{{\text{pkm}}\;\left({{\text{e.g.}}\;{\text{cars}}} \right)}}{{\text{country-specific}\;{\text{occupancy}}\;{\text{rate}}\left( {{\text{e.g.}}\;{\text{cars}}} \right)}} $$

We estimated the vkm for bikes by multiplying the average distance (in km) cycled per capita per day (data collected between 2008 and 2015, from Steenberghen et al. 2017), by 365 and by the countries’ population in 2017. Our estimate is limited to 13 countries (Austria, Belgium, Cyprus, Denmark, Finland, France, Germany, Ireland, Latvia, Netherlands, Slovakia, Sweden, and United Kingdom), because data were not available for the remaining 15 countries. The countries that are included represent 56% of the EU’s population.

#### Tyre use

The amount of natural rubber consumed during vehicle use is determined by tyre use, which depends on the number of wheels per vehicle and the lifespan of a tyre (Table S2). Values for tyre lifespan were obtained from TNO (2016) and USTMA (2020a) for passenger cars and light vans, from ETRMA (2017) for buses and trucks, and from Schwalbe (2021) for motorcycles and bikes, while the number of wheels per vehicle was estimated based on the vehicles’ structure. We estimated tyre use per category of vehicle following Eq. (2).

2 $$ {\text{tyre}}\,{\text{use}}\;\left({{\text{e.g.}}\;{\text{cars}}}\right) = \frac{{{\text{vkm}}\;\left({{\text{e.g.}}\;{\text{cars}}}\right)}}{{\text{tyre}\;{\text{lifespan}}\;\left({{\text{e.g.}}\;{\text{car}}}\right)}} \times {\text{number}}\;{\text{of}}\;{\text{wheels}}\;\left({{\text{e.g.}}\;{\text{per}}\;{\text{car}}} \right) $$

Additionally, we calculated annual tyre use for aircrafts following Eq. (3), using the number of passenger aircrafts registered in each country in 2018 (DG MOVE 2018) and data regarding the number, composition and lifespan of aircraft tyres (the average number of landings before a tyre is replaced) (personal communication, 2019). Although the variety of aircrafts is high, we assumed Embraer 190 to be representative for passenger aircrafts with 150 seats or less and freight aircrafts with maximum take-off weight allowed includes aircraft and cargo weight (mtow) under 45 metric tons, and Airbus A330-200 to be representative for passenger aircrafts with more than 150 seats and freight aircrafts with mtow over 45 metric tons. These two aircraft models are widely used in Europe (personal communication, 2019). We estimated the annual number of landings per aircraft from flight records for the year 2019 (Flightradar24 2021).

3 $$ {\text{tyre}}\,{\text{use}}\,\left({\text{aircrafts}}\right) = {\text{number}}\,{\text{of}}\,{\text{aircrafts}} \times ~\frac{{\text{number}\,{\text{of}}\,{\text{landings}}\,{\text{per}}\,{\text{year}}}}{{\text{tyre}}\,{\text{lifespan}}} \times {\text{number}}\,{\text{of}}\,{\text{wheels}}\,\left({{\text{per}}\,{\text{aircraft}}} \right) $$

#### Natural rubber (NR) use

In addition to the quantities of tyres used annually, the natural rubber content of tyres determines the demand for natural rubber. Tyre weight and composition are custom-designed to meet the needs of each vehicle model (USTMA, c2020), but such quantitative and detailed data are scarce. We used the most reliable data we could find into tyre manufacturers’ booklets from several world regions, blogposts, e-shops, rubber compound patents, and communication with experts (Table S2). We are confident that combining data from different years is not problematic since the substitution of natural and synthetic rubbers in industrial products and processes is practically challenging and unprofitable (Wagner 2020). We calculated natural rubber use following Eq. (4). The rubber content (in kg) in tyres is estimated by categories of land vehicles from market data for the year 2017 (JATMA 2019), indicating the number of tyres and tubes produced and the equivalent in tons of rubber. The proportion of natural rubber in passenger and light truck tyres is reported by USTMA (2020b), as well as in truck tyres, which we applied to buses and medium/heavy trucks tyres. In line with the composition of the rubber compound of standard motorcycle tyres described by Ngeow et al. (2013), we assumed 100% of synthetic rubber in motorcycle tyres, but also quantified the maximum impact with 100% natural rubber. For bike tyres, we used the weight and composition indicated for three bestseller models from Decathlon (2021). For aircraft tyres, the natural rubber content equals the weight of the tread as 100% of the rubber is natural (personal communication, 2019).

4 $$ {\text{NR}}\,{\text{use}}\,({\text{e.g.}}\,{\text{cars}}) = {\text{tyre}}\,{\text{use}}\,({\text{e.g.}}\,{\text{cars}}) \times {\text{rubber}}\,{\text{content}} \,({\text{e.g.}}\,{\text{in}}\,{\text{car}}\,{\text{tyre}}) \times {\text{proportion}}\,{\text{NR}}\,({\text{e.g.}}\,{\text{in}}\,{\text{car}}\,{\text{tyre}})$$

### Natural rubber origin and land footprint

Natural rubber is used in a wide range of products other than tyres and is traded in several forms between the countries that harvest, transform or ultimately use it. We translated bilateral trade flows of rubber goods and new vehicles into metric tons of natural rubber, and used an input–output approach to trace back the origin of the natural rubber ultimately used in European countries (Kastner et al. 2011).

#### Metric tons of natural rubber

We used global data on bilateral trade flows (import) from the United Nations (2013) COMTRADE database. We multiplied the traded quantities by the proportion of natural rubber in representative items for 17 categories of rubber goods (Table S3)**,** based on the rubber compounds detailed in Chandrasekaran (2007), Nocil Limited, (2010), and USTMA (n.d.) (Table S4). Additionally, we translated bilateral trade flows of new vehicles with tyres (Table S3) because these are commonly traded with tyres. We used a sample of bilateral flows reported both in metric tons and in number of vehicles to estimate the average weight of a vehicle from each category, and used this weight to convert all bilateral flows into number of vehicles. Then, we multiplied the bilateral flows by a standard number of wheels per vehicle and by the share of natural rubber in a tyre to obtain bilateral flows in metric tons of natural rubber. To account for the volatility in bilateral trade relationships, we used an average of records for 2016, 2017, and 2018. For Slovenia, we excluded values for 2017 that were unusually high.

#### Land footprint in rubber growing countries

We used an input–output approach to trace back the origin of the natural rubber finally used in the EU, following the method developed by Kastner et al. (2011). We used natural rubber production data from FAOSTAT (2019) for 2016. In the absence of explicit data on preferences for natural rubber from specific origins for specific goods, we assumed exports and domestic final consumption to originate proportionally from domestic production and imports, similarly to Kastner et al. (2011). We obtained the natural rubber sourcing matrix for the EU (see Fig. S5), indicating the contribution of each rubber growing country (rows) to the total amount of natural rubber available for final use in each EU country (columns), in proportions, and used it to allocate the EU’s natural rubber use in tyres calculated in Sect. 2.1., among the rubber growing countries (Fig. 1). The land footprint of EU mobility in 2017 is then estimated using country-specific yield data from FAOSTAT (2019) for 2016.

### Drivers of natural rubber use in the European Union

First, we compared the rubber efficiency of overland vehicles used for passenger and freight transport to reflect on the performance of different vehicle choices in the EU. Second, we assessed how car use for different purposes influences the average amount of natural rubber used per capita in 2017, using indicators available for all EU 28 countries.

#### Rubber efficiency of vehicles

Passenger cars, buses, motorcycles, bikes, and passenger aircrafts are assumed to serve personal mobility, while light vans, medium and heavy trucks, and freight aircrafts are assumed to serve the transport of goods. Each type of vehicle uses a different amount of natural rubber to serve personal mobility or the transport of goods. To assess how the preference for certain types of vehicle influences demand for natural rubber, we compared the rubber efficiency of vehicles, i.e. the amount of natural rubber used to transport one person or one metric ton of goods over 100 km, on average, in the EU.

#### Purposes of car use

Cars shape people’s lifestyles by supporting personal transport but also by serving as a means of expressing a certain lifestyle (Buehler et al. 2017). The car is currently the most widely used mode of transport in the EU (Pastori et al. 2018) for everyday travel and for long-distance trips for business or leisure (Aparicio 2016). To assess how these three main purposes of car use influence demand for natural rubber, we used country-level indicators for the EU 28 derived from data from DG MOVE (2014) and Eurostat (2020a, b) for 2017 (Table 1). We used the Spearman's rank correlation, a non-parametric test without assumptions regarding the distribution of the data, to measure the relation between per capita natural rubber use in car tyres in 2017, and the extent to which the car is used for different purposes at the country level. We also estimated correlations between car use for different purposes to understand co-occurrence.

**Table 1:** Indicators of car use for three main purposes at the country level

Purposes of car use	Indicator	Data source
Everyday travel, e.g. to and from work, housekeeping and leisure activities	Share of the population using the car as main mode of transport for everyday travel	DG MOVE (2014) for 2014
Travel to professional gatherings that involve at least one overnight stay	Number of business trips by car per capita	Eurostat (2020a) for 2017
Travel to vacations that involve at least one an overnight stay	Number of holiday trips by car per capita	Eurostat (2020) for 2017

## Results

### Vehicles, tyres and natural rubber use in the European Union

Two-thirds of the total distance travelled in 2017 by overland vehicles with tyres registered in the EU is covered by passenger cars, while freight transport accounts for 17% of which 70% is comprised by light vans (Table 2). Passenger cars also dominate the demand for tyres (55%). Motorcycles and bikes each constitute around 16% of the total demand for tyres, due to their lower average tyre lifespan (Table S2). Conversely, the relatively longer lifespan of truck and bus tyres compensates for the higher number of wheels when transforming distance into number of tyres used. We estimated that around 672 000 metric tons of natural rubber are consumed annually by the vehicles with tyres registered in the EU, corresponding to 5% of global natural rubber production (ETRMA 2019a). Nearly two-thirds of this amount is used for personal mobility, and one-third to move goods. Major contributors are passenger cars and light vans, due to long distances travelled, and trucks due to the high share of natural rubber in their tyres. Motorcycles do not contribute to natural rubber consumption as long as their tyres do not contain any natural rubber. Assuming 100% natural rubber in motorcycle tyres would increase their contribution to total natural rubber use to an amount similar to that of cars (Table 2). Aircrafts account for only 0.06% of the natural rubber consumption, even though the rubber used in aircraft tyres is 100% natural, and frequently renewed.

**Table 2:** Contribution of vehicles with tyres used for personal mobility and transport of goods, to the total distance travelled, the number of tyres used, and the quantity of natural rubber used in a year in the EU 28. We considered bike use in 13 countries, representing 56% of the EU28 population, and did not assess the distance travelled by aircrafts

Vehicle category	Distance (10^9^ vkm)	Tyres used (10^6^ units)		Natural rubber (10^3^ metric tons)		Share of the total natural rubber use (%)	
		Mid value	Min–max	Mid value	Min–max	Under mid value	Under min–max values
Passenger cars	3279	202	101–262	391	195–508	58%	50%–32%
Buses	28	0.8	0.5–2	14	9–41	2%	2%–6%
Motorcycles	157	63	31–157	0	0–486	0%	0%–30%
Bikes (13 countries only)	144	58	29–144	17	5–99	3%	1%–6%
Passenger aircraft	NA	0.8	0.8–0.8	0.4	0.4–0.4	0.06%	0.1%–0.03%
Personal mobility	3609	325	162–566	422	209–1134	63%	53%–74%
Light vans	511	31	16–41	82	41–106	12%	11%–7%
Medium and heavy trucks	223	10	8–21	168	136–359	25%	35%–22%
Freight aircrafts	NA	0.1	0.1–0.1	0.04	0.04–0.04	0.006%	0.01%–0.0003%
Transport of goods	734	41	24–62	250	177–465	37%	46%–29%
Total	4342	366	186–628	672	384–1599	100%	100%

### Origin of natural rubber used in the EU 28 and respective land footprint

**Fig. 2:**
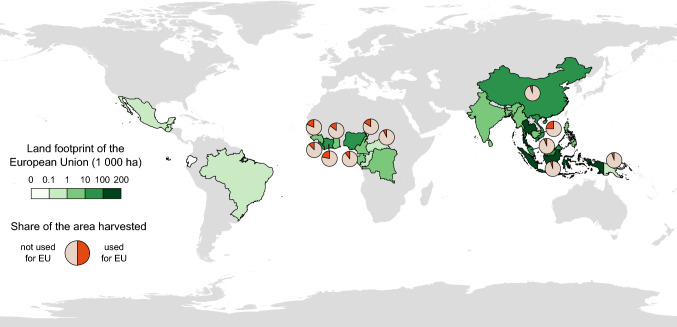
Spatial distribution of the land footprint of mobility in the EU. For countries where the EU uses > 5% of the harvested area, pie charts show the share of the total area harvested in 2016 used to support mobility in the EU in 2017

An area of 594 000 hectares is required to produce the natural rubber consumed annually through tyre use in the EU, corresponding to 5% of the total global area harvested annually (Table S5). This land footprint is mainly located in Indonesia (32%), Thailand (23%), Malaysia (11%), and Côte d’Ivoire (10%), and China (11%) (Fig. 2). At the national level, the share of land harvested to produce tyres for use in the EU is particularly high in Cambodia (25%) and several African countries. In Côte d’Ivoire, Guinea, and Cameroon, more than 15% of the area under mature rubber plantations serves European mobility (Fig. 2).

### Rubber efficiency of overland transport

**Fig. 3:**
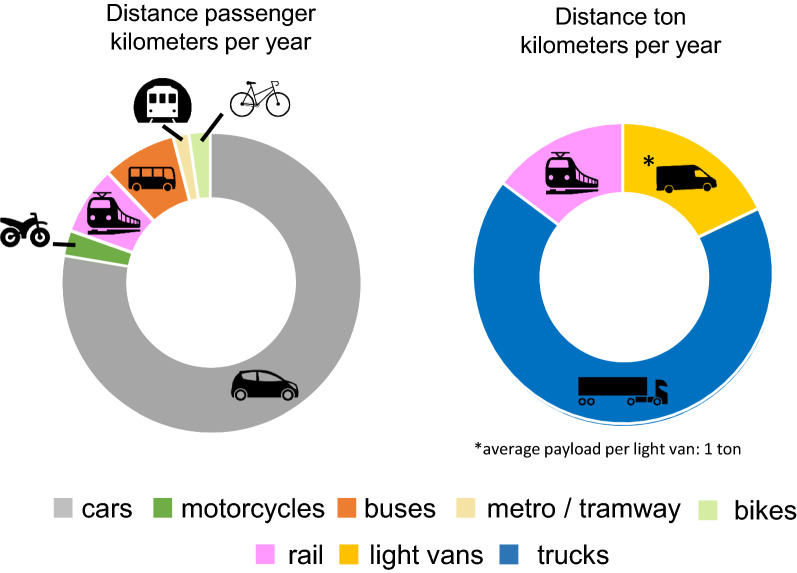
Modal share of overland vehicles used for personal mobility (left chart) and for transport of goods (right chart) in the EU according to the total distance travelled in pkm, and tkm

More than three-quarters of the distance travelled overland in the EU in 2017 was by car. The remaining quarter was shared among buses (8% of total pkm), rail (7%), motorcycles, bikes (estimation limited to 13 countries), and public transport (less than 1% each). The overland transport distance of goods (in tkm) mainly involved trucks with a payload over 3.5 metric tons (two-thirds), followed by vans (18%) and rail (15%) (Fig. 3).

**Fig. 4:**
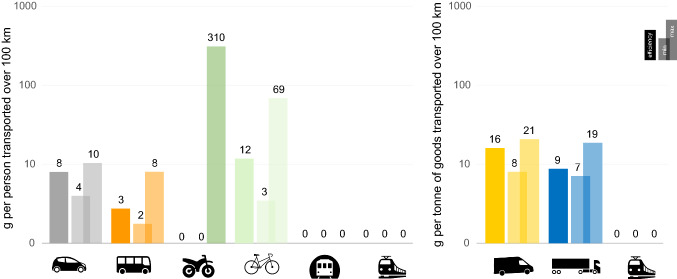
Comparison of the rubber efficiency of overland vehicles (in g per 100 km), between those used for personal mobility (left), and between those used to transport goods (right)

In terms of natural rubber consumption, bikes are less efficient than cars for personal mobility, but this varies according to the type of bike used (Table S2), i.e. road bikes, which are the most convenient for long distances, are more efficient (Fig. 4). Cars are less efficient than buses because of their low occupancy and fast tyre turnover. The efficiency of motorcycles relies on the high proportion of synthetic rubber in the tyres. Metros, tramways, or trains do not use natural rubber for personal mobility, or for the transport of goods. Vans consume more natural rubber than trucks to transport one metric ton of goods over 100 km (Fig. 4).

### Car use

**Fig. 5:**
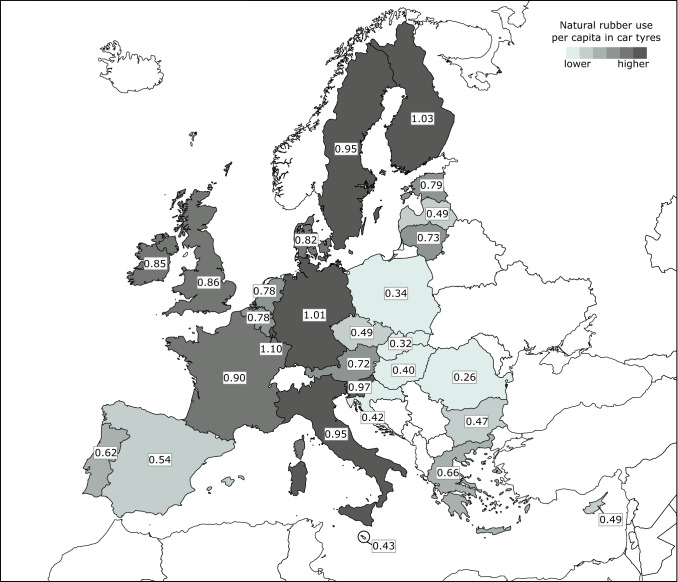
Per capita natural rubber use (in kg) in car tyres in countries of the EU in 2017

Per capita natural rubber use in car tyres varies strongly among European countries (Fig. 5). The highest consumption in 2017 is observed in Luxembourg (1.10 kg) and the lowest in Romania (0.26 kg). Higher levels of consumption are generally observed in wealthier countries with a relatively high motorization rate (Figure S2).

At the national level, per capita use of natural rubber in car tyres is positively correlated with national indicators reflecting the habit of using the car for everyday trips or long-distance travel for business and leisure (Table 3, Fig. S2). The strongest correlation (0.83) is found between per-capita natural rubber use and the share of population commuting by car. Weaker, yet positive correlations are found between per-capital natural rubber use and number of business and leisure trips by car per year per capita. Correlations between indicators of car use for different purposes are also positive, and often stronger than correlations with per capita use of natural rubber (Table 3).

**Table 3:** Spearman-Rank correlations between per capita use of natural rubber in car tyres and the extent to which the car is used for three main purposes. *P* value: **0.01, ***0.001

	Share of the population using the car for most everyday travel	Number of business trips by car per capita per year	Number of leisure trips by car per capita per year
Per capita annual use of natural rubber in car tyres	0.83***	0.60***	0.49**
Share of the population using mostly the car for everyday travel		0.66***	0.58**
Number of business trips by car per capita per year			0.72***

## Discussion

### Interpretation of the results

While the consequences of large-scale conversion of forested land into monoculture rubber plantations are increasingly documented, the demand for natural rubber, the main driver of these land use conversions, remains underexplored. Rapid, recent developments in China are often mentioned as a major driver of the rubber boom (e.g. Qiu 2009; Warren-Thomas et al. 2015), whereas mobility patterns in Western societies are rarely addressed. Our study demonstrates that mobility in the EU uses nearly a fifth of the annual harvest of natural rubber in several producing countries, therefore contributing to the expansion of rubber plantations in the tropics (Fig. 2). Attributing the use of natural rubber to vehicle categories shows that natural rubber use is mainly related to car use (Table 2), underlining the role of personal mobility. We found large differences in the per capita natural rubber use in car tyres among countries of the EU, with highest values in more affluent countries (Fig. 5). This suggests that demand from the EU might increase with economic development in the eastern member states in the near future (Tsemekidi Tzeiranaki et al. 2020).

Clear differences in the rubber efficiency of overland vehicles (Fig. 4) suggest that modal shifts can contribute to decreasing the demand for natural rubber, but the substitutability of different overland vehicles might be limited or inefficient in practice. Vans are more convenient to transport goods over short distances with frequent stops, e.g. in urban centres (Browne et al. 2010) or for e-commerce deliveries for which vans might also substitute the use of private cars for shopping-related travel (Rotem-Mindali and Weltevreden 2013). Reducing the demand for natural rubber by favouring air freight is not desirable because it can increase carbon emissions. Transport by inland waterways is a rubber and carbon efficient option, but relies on climatic conditions (Sims et al. 2015) and availability of navigable waterways. Low average car occupancy rates (< 2) in most European countries (Fiorello and Zani 2018) explains car inefficiency for personal mobility. In low-density areas, where collective alternatives to the car for commuting are limited, shared mobility could be an option (Sims et al. 2015). Nevertheless, even with rising occupancy rates, cars are unlikely to outperform public transport (Fig. S1). In urban centres, the promotion of bike use remains a sustainable option because it avoids energy consumption for mobility, but emerging trends, such as the use of electric fat bikes for everyday travel, could turn out to be a detrimental shift.

National indicators revealed that natural rubber use is related to the widespread use of cars for everyday travel, which in turn is associated with relatively frequent long-distance travel by car (Table 3). Car ownership is the strongest driver for frequent car use, ahead of commuting distance or access to public transport infrastructure (Focas and Christidis 2017). Motorization rates have been shown to increase with disposable income because, beyond its practicality, the car confers social status and gives access to symbolic activities such as leisure travel (Steg et al. 2001). Additionally, many Europeans receive a company car with a discounted access to fuel for business and personal travel (Gutiérrez i Puigarnau and van Ommeren 2011), which results in a significant level of additional mileage (Shiftan et al. 2012). Conversely, the use of private cars can be limited due to operating costs (e.g. Poland, Czech Republic) or the preference for soft modes for everyday travel (e.g. Austria) (Haustein and Nielsen 2016) (Fig. S2).

### Limitations

There is a lack of official, openly available data on most aspects related to natural rubber use and production. We therefore had to rely on a wide range of data from hugely different sources. As a consequence, uncertainties in some parameters are high (See Tables S2, S5, S6, and Fig. 4). Tyre lifespan estimates vary by a factor two, perhaps revealing the diversity of models that differ in size and composition (ASDA 2019), to match different vehicles and ensure safety on the road under all weather conditions (Uniroyal 2018). Truck tyres “made in Europe” have a greater lifespan than imported tyres, because they are built to be retreaded up to three times (ETRMA 2017b). Over the last decade, EU’s imports of truck tyres, especially from China, have increased (ETRMA 2017a), but the share of imported tyres in total use is uncertain as tyres can be re-exported. In addition, the number of wheels per bus and truck varies with the type of vehicle but also with the load transported. When combining minimum numbers of wheels with maximum tyre lifespan, the contribution of personal mobility and transport of goods to natural rubber use for mobility in the EU are almost equal, and the land footprint is reduced to 342 000 ha. In the reverse scenario, the annual use of natural rubber reaches 1.6 million metric tons, including 486 000 metric tons used in motorcycle tyres, and the land footprint reaches 1.4 million ha, corresponding to 12% of the total area harvested in 2016. Finally, the contribution of bike use to natural rubber use in the EU is likely to be largely underestimated because data on the annual distance travelled were missing for 15 countries, and the contribution of e.g. city, road, all-terrain bikes to this distance is unknown. Accounting for missing countries would also likely increase the total land footprint.

We quantified natural rubber use through tyre use, but natural rubber is also used in small amounts in other vehicle parts (e.g. train car junctions, passenger car mats), meaning that transportation uses more than the estimated 70% of global natural rubber production. To quantify the land footprint, we used production and yield data from FAOSTAT (2019), because it is the only available comprehensive free global data source. However, a large proportion of these data is imputed by FAOSTAT (2019) because producing countries do not consistently report them (Table S8). Country-specific yields are particularly uncertain because they depend on planting and tapping schemes, which are not officially monitored. Natural rubber can have a wide range of exact specifications, and exporting countries might specialize in producing natural rubber for specific goods (e.g. tyres, industrial parts). However, we calculated the contribution of each producing country based on reported trade flows that only provide an aggregated figure for natural rubber and do not allow for distinguishing between specific products. Moreover, by applying the approach from Kastner et al. (2011) we included the main assumption that the natural rubber contained in exported items originates proportionally from import and production. While these two assumptions impact the distribution of the land footprint, our estimate that tyre use in the EU uses around 5% of global natural rubber production is in line with ETRMA (2019b), who report that the EU ultimately uses 9% of the natural rubber produced in the world, of which 70% is used in tyres. In addition, the EU's strategy to reduce its dependence on Southeast Asia (ETRMA 2019b) is reflected in our estimate that almost 25% of the natural rubber imported in Europe originates from Africa, which is more than Africa's contribution to global production (FAOSTAT 2019).

### Implications

Political and economic decisions in recent decades have shaped urban planning and people's preferences for private cars (Mattioli et al. 2020). Given that almost 75% of Europeans live in cities, suburbs and towns (Eurostat 2016), the potential impact of urban planning on reducing car use for commuting and the EU's land footprint in tropical countries is high. Pricing measures targeting road and fuel use, and the expansion of cycling and walking lanes at the expense of parking and traffic space in congested cities, can encourage a modal shift (Sims et al. 2015). When economic stress is low, maintaining high car ownership rates does not prevent a reduction in car use for everyday travel (e.g. the Netherlands (Kroesen 2017)), but might result in an upturn in the car mileage for long-distance travel (Holz-Rau et al. 2014). To reduce the number of car owners, functional and symbolic aspects of passenger cars must be addressed (Axsen and Sovacool 2019). Environmental awareness can be a trigger, but habits (Sopjani et al. 2020) and active lifestyles (Haustein and Nielsen 2016), often involving high mobility to reconcile work and private life (Viry and Kaufmann 2015), are barriers. The Covid-19 pandemic has reduced travel opportunities, but its impact on car ownership is uncertain, as private cars allow for traveling while limiting social contacts (Kanda and Kivimaa 2020).

Solutions to limit the consumption of natural rubber result in synergies and trade-offs with the main objective of the Paris Agreement to drastically and rapidly lower global greenhouse gas emissions levels. Car use is responsible for around 12% of the EU’s carbon emissions (DG Climate 2011). A modal shift towards collective alternatives for overland personal mobility could reduce carbon emissions and pressure on land for rubber, as the emission intensity of cars is higher (Sims et al. 2015; Ivanova et al. 2018). Conversely, replacing the EU’s car fleet with electric cars is expected to decrease carbon emissions by up to 70% by 2050 (Lutsey 2015), but will not lower demand for natural rubber or non-exhaust particulate emissions from the wearing down of tyres (OECD 2020). Measures to regulate carbon emissions from air transport (Sims et al. 2015) might trade off into increasing use of natural rubber if passenger and freight transport is not reduced or taken over by rail and waterway transport.

The EU’s ambition to reduce the transport sector’s dependence on oil, in order to prevent the consequences of its scarcity on the competitiveness of the European economy (European Commission 2011), might increase rubber demand. Synthetic rubber is derived from mineral oil and widely used to manufacture tyres (e.g. 56% of the rubber used in a passenger car tyre) (USTMA, c2020b). For the time being, the properties of synthetic rubber are essential for some tyre components (Millard 2019), but tyre manufacturers have already started to work on overcoming barriers to substitutability between synthetic and natural rubber (Krok 2017). Full replacement of synthetic rubber with natural rubber in all tyres would more than double the land footprint of mobility in the EU (Table S7), rendering a risk of carbon loss to the atmosphere through deforestation. A policy aiming at synthetic rubber substitution should carefully consider and avoid such trade-offs.

Demand projections are a factor influencing the expansion of rubber plantations. Given that the EU is willing to reduce its dependency on Southeast Asia (ETRMA 2019b), increasing demand might stimulate the expansion of rubber in other producing regions. In Central Africa, for instance, foreign investment in agro-industrial plantations is welcome, threatening vast areas of forested land that are often not adequately protected by governments (Feintrenie 2014). In addition, the ecological footprint of tyre use in the EU could extend beyond the tropics, as the EU is involved in research on the use of natural rubber produced from plants that can be grown at other latitudes (Cornish 2017).

Sustainability standards in natural rubber production are lagging behind those of other commodities (Millard 2019) because initiatives from the tyre industry are difficult to implement due to the large number of small producers (Warren-Thomas et al. 2015). Since 2019, however, a Global Platform for Sustainable Natural Rubber (GPSNR 2020) has been working towards managing sustainability in the supply chain, with particular attention to the long-term effects of production practices. To that respect, agroforestry systems can reduce the negative impacts of natural rubber production on biodiversity (Langenberger et al. 2017), but the land footprint tied to mobility can only be limited if the demand for natural rubber levels-off or decreases.

## Conclusion

Our quantification of natural rubber use in tyres has shown that mobility in the European Union in 2017 required around 5% of global rubber production area in 2016. Preference for road vehicles over other overland options for personal mobility and transport of goods drives the EU’s natural rubber consumption. Car tyres account for 58% of Europe’s annual use of natural rubber, highlighting the importance of personal mobility. This importance however varies across the EU, with annual per capita values ranging from 0.26 kg in Romania to 1.10 kg in Luxembourg. High values are related to the predominant use of cars for everyday trips and frequent long-distance travel by car. Car travel is the norm in many European countries where car ownership and economic prosperity are high and spatial planning has produced car-dependent lifestyles. Daily car use triggers people to also use their car for long-distance business and leisure travel, which tend to be more frequent in countries with high per capita income. Addressing car dependency is therefore key to reducing future demand for rubber, avoiding pressures on tropical lands which otherwise risk conversion to rubber plantations. Policy action that cuts across the domains of transport, spatial planning, trade, and climate change is required to both address the multiple drivers of car use, and avoid trade-offs between natural rubber use and carbon emissions.

## Supplementary Information

Below is the link to the electronic supplementary material.

Supplementary file1 (PDF 772 kb)

## References

[CR1] Ahrends A, Hollingsworth PM, Ziegler AD, Fox JM, Chen H, Su Y, Xu J (2015). Current trends of rubber plantation expansion may threaten biodiversity and livelihoods. Global Environmental Change.

[CR2] Aparicio A (2016). Exploring recent long-distance passenger travel trends in Europe. Transportation Research Procedia.

[CR3] ASDA. 2019. The difference between summer and winter tyres [blog]. https://www.asdatyres.co.uk/blog/the-difference-between-summer-and-winter-tyres/.

[CR4] Axsen J, Sovacool BK (2019). The roles of users in electric, shared and automated mobility transitions. Transportation Research Part D: Transport and Environment.

[CR5] Barnes AD, Jochum M, Mumme S, Haneda NF, Farajallah A, Widarto TH, Brose U (2014). Consequences of tropical land use for multitrophic biodiversity and ecosystem functioning. Nature Communications.

[CR6] Berkelmann D, Schneider D, Meryandini A, Daniel R (2020). Unravelling the effects of tropical land use conversion on the soil microbiome. Environmental Microbiome.

[CR7] Browne M, Allen J, Nemoto T, Visser J (2010). Light goods vehicles in urban areas. Procedia - Social and Behavioral Sciences.

[CR8] Buehler R, Pucher J, Gerike R, Götschi T (2017). Reducing car dependence in the heart of Europe: Lessons from Germany, Austria, and Switzerland. Transport Reviews.

[CR9] Chandrasekaran. 2007. *Essential rubber formulary. Formulas for practitioners*, ed. S. Ebnesajjad. William Andrew Publishing.

[CR10] Cornish K (2017). Alternative natural rubber crops: Why should we care?. Technology & Innovation.

[CR11] Debbarma R, Purkayasthaî S (2019). Expansion of area under rubber plantation and its distribution in Tripura, India. Space and Culture India.

[CR12] Decathlon. 2021. Bike tires. https://www.decathlon.com/collections/bike-tires.

[CR13] DG Climate. 2011. Reducing CO2 emissions from passenger cars. www.ec.europa.eu/clima/policies/transport/vehicles/cars/index_en.

[CR14] DG MOVE. 2014. *Special Eurobarometer 422a “Quality of transport.”*10.1787/eco_surveys-grc-2013-graph14-en.

[CR15] DG MOVE. 2018. *Statistical pocketbook 2018. Connecting Europe. Mobility and Transport*. *Publications Office of the European Union*. 10.2832/05477.

[CR16] Dove MR (2018). Rubber versus forest on contested Asian land. Nature Plants.

[CR17] ETRMA. 2017a. European type and rubber industry, statistics.

[CR18] ETRMA. 2017b. *Moving innovation that cares*. *Annual Report*. Brussels. http://www.etrma.org/uploads/Modules/Documentsmanager/20170905---etrma-annual-report-2016-17---final.pdf.

[CR19] ETRMA. 2019a. European type and rubber industry, statistics, 48. https://www.etrma.org/wp-content/uploads/2019/10/20191114-Statistics-booklet-2019-Final-for-web.pdf.

[CR20] ETRMA. 2019b. Sustainable Natural Rubber & European Commission Deforestation Agenda. https://ec.europa.eu/environment/forests/pdf/respondents-additional-inputs/EuropeanTyre and Rubber Manufacturers%27Association (ETRMA).pdf.

[CR21] European Commission. 2011. White Paper. https://ec.europa.eu/info/sites/info/files/commission-white-paper-artificial-intelligence-feb2020_en.pdf.

[CR22] Eurostat. 2016. *Urban Europe. Statistics on cities, towns and suburbs. 2016 edition*. *The European Territory*. 10.4324/9781315777962-2.

[CR23] Eurostat. 2020a. Eurostat Statistical database. https://ec.europa.eu/eurostat/data/database.

[CR24] Eurostat. 2020b. Eurostat Statistical database.

[CR25] FAOSTAT. 2019. FAO Statistical Database. http://faostat.fao.org/.

[CR26] Feintrenie L (2014). Agro-industrial plantations in Central Africa, risks and opportunities. Biodiversity and Conservation.

[CR27] Fiorello, D., and L. Zani. 2018. Car occupancy rates in the European Union. From *EU Survey on issues related to transport and mobility*. European Commission. Joint Research Center. Institute for Prospective techonological studies. Obtained on request.

[CR28] Flightradar24. 2021. Live air traffic. https://www.flightradar24.com/.

[CR29] Focas C, Christidis P (2017). What drives car use in Europe?. Luxembourg.

[CR30] Fox J, Castella JC, Ziegler AD (2014). Swidden, rubber and carbon: Can REDD+ work for people and the environment in Montane Mainland Southeast Asia?. Global Environmental Change.

[CR31] Gov.UK. 2013. A guide to lorry types and weights [blog]. https://www.gov.uk/government/publications/guide-to-lorry-types-and-weights. Accessed 15 Sept 2019.

[CR32] GPSNR. 2020. Global platform for sustainable natural rubber. https://sustainablenaturalrubber.org/.

[CR33] Grogan K, Pflugmacher D, Hostert P, Mertz O, Fensholt R (2019). Unravelling the link between global rubber price and tropical deforestation in Cambodia. Nature Plants.

[CR34] Gutiérrez i Puigarnau, E., and J.N. van Ommeren (2011). Welfare effects of distortionary fringe benefits taxation: The case of employer-provided cars. International Economic Review.

[CR35] Haustein S, Nielsen TAS (2016). European mobility cultures: A survey-based cluster analysis across 28 European countries. Journal of Transport Geography.

[CR36] Heer K, Helbig-Bonitz M, Fernandes RG, Mello MAR, Kalko EKV (2015). Effects of land use on bat diversity in a complex plantation-forest landscape in Northeastern Brazil. Journal of Mammalogy.

[CR37] Holz-Rau C, Scheiner J, Sicks K (2014). Travel distances in daily travel and long-distance travel: What role is played by urban form?. Environment and Planning A.

[CR38] Hurni K, Fox J (2018). The expansion of tree-based boom crops in mainland Southeast Asia: 2001 to 2014. Journal of Land Use Science.

[CR39] ITF. 2019. *ITF transport outlook 2019*. Paris. 10.1787/transp_outlook-en-2019-en.

[CR40] Ivanova D, Vita G, Wood R, Lausselet C, Dumitru A, Krause K, Macsinga I, Hertwich EG (2018). Carbon mitigation in domains of high consumer lock-in. Global Environmental Change.

[CR41] JATMA. 2019. *Tyre industry of Japan* , vol. 18. https://www.jatma.or.jp/media/pdf/tyre_industry_2019.pdf.

[CR42] Kanda W, Kivimaa P (2020). What opportunities could the COVID-19 outbreak offer for sustainability transitions research on electricity and mobility?. Energy Research and Social Science.

[CR43] Kastner T, Kastner M, Nonhebel S (2011). Tracing distant environmental impacts of agricultural products from a consumer perspective. Ecological Economics.

[CR44] Kroesen M (2017). To what extent do e-bikes substitute travel by other modes? Evidence from the Netherlands. Transportation Research Part D: Transport and Environment.

[CR45] Krok, A. 2017. GM wants all its tires to contain natural, sustainable rubber [blog]. https://www.cnet.com/roadshow/news/gm-wants-all-its-tires-to-contain-natural-sustainable-rubber/?sf79234876=1.

[CR46] Lang CR (2001). Deforestation in Vietnam, Laos, and Cambodia. Deforestation, environment, and sustainable development: A comparable analysis.

[CR47] Langenberger G, Cadisch G, Martin K, Min S, Waibel H (2017). Rubber intercropping: A viable concept for the 21st century?. Agroforestry Systems.

[CR48] Liu CA, Nie Y, Zhang YM, Tang JW, Siddique KHM (2018). Introduction of a leguminous shrub to a rubber plantation changed the soil carbon and nitrogen fractions and ameliorated soil environments. Scientific Reports.

[CR49] Lutsey N (2015). Global climate change mitigation potential from a transition to electric vehicles | International Council on Clean Transportation. The International Council on Clean Transportation.

[CR50] Mattioli G, Roberts C, Steinberger JK, Brown A (2020). The political economy of car dependence: A systems of provision approach. Energy Research and Social Science.

[CR51] Meyer, B. 2019. Natural rubber prices remain cyclical [blog]. https://www.rubbernews.com/suppliers/natural-rubber-prices-remain-cyclical. Accessed 20 Nov 2019.

[CR52] Millard E, Schmidt M, Giovannucci D, Palekhov D, Hansmann B (2019). Recent experiences from the natural rubber industry and its movement towards sustainability. Sustainable global value chains.

[CR53] Neyret M, Robain H, de Rouw A, Janeau JL, Durand T, Kaewthip J, Trisopon K, Valentin C (2020). Higher runoff and soil detachment in rubber tree plantations compared to annual cultivation is mitigated by ground cover in steep mountainous Thailand. CATENA.

[CR54] Ngeow, Y.W., M. Mustapha Kamal, P.C. Khaw, A.K. Che Aziz, and T.Z. Zaeimoedin. 2013. *WO 2013/172699 Al*. Malaysia. https://patentscope.wipo.int/search/docs2/pct/WO2013172699/pdf/c2QdL6C4PzTd9zX0ME8-G5UixNaczbmpMcXIcAeGRBujpSVTAGNoKPLXrcsJoqKabNBYv_FZV8_B4YFOCQtXgPJjgkecV4Ovxextc2bFJDxQonEG2xY7qh1tW2ZwsuHP?docId=id00000023073338.

[CR55] Nocil Limited. 2010. Starting point rubber compounding formulations, 1–35. http://www.nocil.com/Downloadfile/CCompoundingFormulations&UsefulInfo-Dec2010.pdf.

[CR56] OECD. 2020. *Non-exhaust particulate emissions from road transport: An ignored environmental policy challenge.* Paris. 10.1787/4a4dc6ca-en.

[CR57] Ouattara, O. 2019. *Diffusion de l’hévéaculture et sécurité alimentaire en Côte d’Ivoire: approche dans les régions de l’Indénié-Djuablin et de la Nawa*. Université de Nantes.

[CR58] Paoletti A, Darras K, Jayanto H, Grass I, Kusrini M, Tscharntke T (2018). Amphibian and reptile communities of upland and riparian sites across Indonesian oil palm, rubber and forest. Global Ecology and Conservation.

[CR59] Pastori, E., M. Brambilla, S. Maffii, R. Vergnani, E. Gualandi, E. Dani, and I. Skinner. 2018. Research for TRAN Committee—Modal shift in European transport: A way forward, (November). http://www.europarl.europa.eu/thinktank/en/document.html?reference=IPOL_STU(2018)629182.

[CR60] Penot, E., B. Chambon, and G. Wibawa. 2017. An history of Rubber Agroforestry Systems development in Indonesia and Thailand as alternatives for a sustainable agriculture and income stability. *Proceedings of International Rubber Conference* (October), 497–532.

[CR61] Qiu J (2009). Where the rubber meets the garden. Nature.

[CR62] Reddy CS, Pasha SV, Jha CS, Diwakar PG, Dadhwal VK (2016). Development of national database on long-term deforestation (1930–2014) in Bangladesh. Global and Planetary Change.

[CR63] Rotem-Mindali O, Weltevreden JWJ (2013). Transport effects of e-commerce: What can be learned after years of research?. Transportation.

[CR64] Schwalbe. 2021. Schwalbe. https://www.schwalbe.com/en/verschleiss.

[CR65] Shiftan Y, Albert G, Keinan T (2012). The impact of company-car taxation policy on travel behavior. Transport Policy.

[CR66] Sims, R., R. Schaeffer, F. Creutzig, X. Cruz-Núñez, M. D’Agosto, D. Dimitriu, et al. 2015. Transport. In *Climate Change 2014: Mitigation of Climate Change. Contribution of Working Group III to the Fifth Assessment Report of the Intergovern- mental Panel on Climate Change*, ed. Edenhofer, O., R. Pichs-Madruga, Y. Sokona, E. Farahani, S. Kadner, K. Seyboth, A. Adler, I. Baum, S. Brunner, P. Eickemeier, B. Kriemann, J. Savolainen, S. Schlömer, and C. von Stechow, 599–670 . Cambridge: Cambridge University Press.

[CR67] Singh D, Slik JWF, Jeon YS, Tomlinson KW, Yang X, Wang J, Kerfahi D, Porazinska DL (2019). Tropical forest conversion to rubber plantation affects soil micro- & mesofaunal community & diversity. Scientific Reports.

[CR68] Sopjani L, Stier JJ, Hesselgren M, Ritzén S (2020). Shared mobility services versus private car: Implications of changes in everyday life. Journal of Cleaner Production.

[CR69] Steenberghen T, Tavares T, Himpe W, Crabbé A (2017). Support Study on Data Collection and Analysis of Active Modes Use and Infrastructure in Europe Final Report..

[CR70] Steg, L., Vlek, C., and Slotegraaf, G. 2001. *Transportation Research Part F: Traffic Psychology and Behaviour*, *4*, 151–169.

[CR71] TNO (2016). Study on some safety-related aspects of tyre use. Helmond.

[CR72] TRACCS. 2013. *Transport data collection supporting the quantitative analysis of measures relating to transport and climate change*, ed. European Commission; DG Climate Action. https://traccs.emisia.com/index.php?type=project.

[CR73] Tsemekidi Tzeiranaki, S., P. Bertoldi, D. Paci, L. Castellazzi, T. Serrenho, M. Economidou, and P. Zangheri. 2020. *Energy consumption and energy efficiency*. *EUR 30328*. Luxembourg. 10.1017/9781108861595.009.

[CR74] Uniroyal. 2018. Where are winter tyres mandatory in Europe? [blog]. https://www.uniroyal-tyres.com/car/tyre-guide/winter-care/winter-tyres-mandatory.

[CR75] United Nations. 2013. UN Comtrade Database.

[CR76] USTMA. 2020a. How is a tire made? https://www.ustires.org/howatireismade.

[CR77] USTMA. 2020b. What is in a tire? https://www.ustires.org/whats-tire-0.

[CR78] Van Straaten O, Corre MD, Wolf K, Tchienkoua M, Cuellar E, Matthews RB, Veldkamp E (2015). Conversion of lowland tropical forests to tree cash crop plantations loses up to one-half of stored soil organic carbon. Proceedings of the National Academy of Sciences of the United States of America.

[CR79] Viry, G., and V. Kaufmann. 2015. *High mobility in Europe*, ed. G. Viry and V. Kaufmann. Palgrave Macmillan. 10.1057/9781137447388

[CR80] Wagner N (2020). Why the prices of natural and synthetic rubber do not always bounce together: Beyond the Numbers: U.S. Bureau of Labor Statistics. US Bureau of Labor Statistics.

[CR81] Warren-Thomas E, Dolman PM, Edwards DP (2015). Increasing demand for natural rubber necessitates a robust sustainability initiative to mitigate impacts on tropical biodiversity. Conservation Letters.

[CR82] Warren-Thomas E, Nelson L, Juthong W, Bumrungsri S, Brattström O, Stroesser L, Chambon B, Penot E (2020). Rubber agroforestry in Thailand provides some biodiversity benefits without reducing yields. Journal of Applied Ecology.

[CR83] WRM. 2020. *What could be wrong about planting trees? The New push for more industrial tree plantations in the global South.* https://stopgetrees.org/what-could-be-wrong-about-planting-trees/.

