# Correction to: Assessing the contribution of mobility in the European Union to rubber expansion

**DOI:** 10.1007/s13280-021-01604-z

**Published:** 2021-07-29

**Authors:** Perrine C. S. J. Laroche, Catharina J. E. Schulp, Thomas Kastner, Peter H. Verburg

**Affiliations:** 1 Institute for Environmental Studies, Vrije Universiteit Amsterdam, De Boelelaan 1087, 1081 HV Amsterdam, The Netherlands; 2 Senckenberg Biodiversity and Climate Research Centre (SBiK-F), Senckenberganlage 25, 60325 Frankfurt-am-Main, Germany; 3 Swiss Federal Research Institute for Forest, Snow and Landscape Research WSL, Birmensdorf, Switzerland

## Correction to: Ambio 10.1007/s13280-021-01579-x

In the original article, the name of a company that did not formally collaborate with the authors of this study has been removed from the acknowledgements and supplementary materials to clear up misinterpretations.

The correct acknowledgement and the supplementary information are provided in this article.

The original article has been corrected.

## Supplementary Information

Below is the link to the electronic supplementary material.

Supplementary file1 (PDF 772 kb)

