# Supplementary material for: Assessing the contribution of mobility in the European Union to rubber expansion
Source: Ambio. 2021 Jun 12;51(3):770–83. doi: 10.1007/s13280-021-01579-x (PMC8197600; doi:10.1007/s13280-021-01579-x)
Supplement: Supplementary file 1 — Supplementary file1 (PDF 772 kb) [file 13280_2021_1579_MOESM1_ESM.pdf]

Ambio

ELECTRONIC SUPPLEMENTARY MATERIAL

This supplementary material has not been peer-reviewed.

Title: Assessing the contribution of mobility in the European Union to rubber expansion

Authors: Perrine C.S.J. Laroche, Catharina J.E. Schulp, Thomas Kastner, Peter H. Verburg

Contact: perrine.laroche@vu.nl

Table S1: Correspondences between the categories of trucks from TRACCS (rows) and EUROSTAT (columns). We used a simplified guide to lorry types and weights from the UK Department of Transports to make the correspondences.

	Lorries - 2 axles .tvkm	Lorries - 3 axles .tvkm	Lorries - 4 axles .tvkm	Lorries - other .tvkm	Lorries and trailers - 2+1 axles .tvkm	Lorries and trailers - 2+2 axles .tvkm	Lorries and trailers - 2+3 axles .tvkm	Lorries and trailers - 3+2 axles .tvkm	Lorries and trailers - 3+3 axles .tvkm	Lorries and trailers - other .tvkm	Road tractors .tvkm	Road tractors and semi-trailers - 2+1 axles .tvkm	Road tractors and semi-trailers - 2+2 axles .tvkm	Road tractors and semi-trailers - 2+3 axles .tvkm	Road tractors and semi-trailers - 3+2 axles .tvkm	Road tractors and semi-trailers - 3+3 axles .tvkm	Road tractors and semi-trailers - other .tvkm
Rigid >3,5 t																	
Rigid <=7,5 t																	
Rigid 7,5 - 12 t																	
Rigid 12 - 14 t																	
Rigid 14 - 20 t																	
Rigid 20 - 26 t																	
Rigid 26 - 28 t																	
Rigid 28 - 32 t																	
Rigid >32 t																	
Articulated 14 - 20 t																	
Articulated 20 - 28 t																	
Articulated 28 - 34 t																	
Articulated 34 - 40 t																	
Articulated 40 - 50 t																	
Articulated 50 - 60 t																	

Table S2: Tyre lifespan, number of wheels, rubber content and proportion of natural rubber in tyres for vehicles with tyres. The values in bold correspond to the values used to achieve the results displayed in the manuscript, while the other values were used as alternatives to calculate the min and max results. Numbers in brackets refer to the data sources listed below the table.

Category of vehicle	Model	Wheel position	Tyre lifespan	Number of wheels per vehicle	Rubber content (kg)	Share of natural rubber (%)
Passenger car			50 000 km (1)<br>**65 000 km** (2)<br>130 000 km (2)	**4**	**4.4** (3)	**44** (9)
Bus			120 000 km (imported single use tyre) (4)<br>**220 000 km** (tyre made in Europe, retreadable) (4)	4 (5)<br>**6** (5)<br>10 (5)	**23** (3)	**76** (9)
Light van			50 000 km (1)<br>**65 000 km** (2)<br>130 000 km (2)	**4**	**5.9** (3)	**44** (9)
Medium and heavy truck (17 categories according to the number of axles on the front and towed vehicles)			120 000 km (imported single use tyre) (4)<br>**220 000 km** (tyre made in Europe, retreadable) (4)	**2 times # of axles on front vehicle** (truck or road tractor) + 2, **3**, 4 **times # of axles on towed vehicle** (trailer or semi-trailer) (7)	**23** (3)	**76** (9)
Motorcycle			2 000 km (6)<br>**5 000 km** (6)<br>10 000 km (6)	**2**	**3.1** (3)	0 (10)<br>**0** (10)<br>100 (10)
Bike			2 000 km (6)<br>**5 000 km** (6)<br>10 000 km (6)	**2**	0.340 (Road bike) (8)<br>**0.365 (City Bike)** (8)<br>0.860 (All terrain) (8)	50 (8)<br>**80** (8)<br>80 (8)
Aircraft =< 150 seats or mtow < 45 tonnes	Embraer 190	Nose	**330 landings** (5)	**2**	**3.3** (5)	**100** (5)
		Main	**630 landings** (5)	**4**	**9.9** (5)	**100** (5)
Aircraft > 150 seats or mtow > 45 tonnes	Airbus A 330-200	Nose	**220 landings** (5)	**2**	**8.6** (5)	**100** (5)
		Main	**260 landings** (5)	**8**	**12.7** (5)	**100** (5)

(1) TNO. (2016). *Study on some safety-related aspects of tyre use*. Helmond. 568 https://doi.org/10.2832/67191
(2) USTMA (c2020). How a Tire is made. https://www.ustires.org/howatireismade. 40 000 miles = 65 000 km, and 80 000 miles = 130 000 km
(3) JATMA. (2019). *Tyre Industry of Japan* (Vol. 18). Retrieved from 485 https://www.jatma.or.jp/media/pdf/tyre_industry_2019.pdf
(4) ETRMA. (2017). *Moving innovation that cares*. *Annual Report*. Brussels. Retrieved from: https://www.etrma.org/wp-content/uploads/2019/09/20170905-etrma-annual-report-2016-17-final.pdf
(5) Personal communication (2019)
(6) Schwalbe. (c2021). Reichshof (DE): Ralf Bohle GmbH; [accessed 2019 Nov 26]. https://www.schwalbe.com/en/verschleiss
(7) Gov.uk. (2013) Jul 15. A guide to lorry types and weights [blog]. Driver and Vehicle Standards Agency. [accessed 2019 Sep 15]. https://www.gov.uk/government/publications/guide-to-lorry-types-and-weights
(8) Decathlon (c2021). https://www.decathlon.com/collections/bike-tires. Models of tyres selected among the bestsellers.
(9) USTMA. (c2020). What is in a tire. https://www.ustires.org/whats-tire-0
(10) Ngeow, Y. W., Mustapha Kamal, M., Khaw, P. C., Che Aziz, A. K., & Zaeimoedin, T. Z. (2013). *WO 2013/172699 Al*. Malaysia. Retrieved from https://patentscope.wipo.int/

Table S3 : UN Comtrade categories of goods considered to source the origin of the natural rubber finally used in Europe.

Code	Description
4001	Natural rubber, balata, gutta-percha, guayule, chicle and similar gums; in primary forms or in plates, sheets or strip
4002	Synthetic rubber and factice derived from oils, in primary forms or in plates, sheets or strip; mixtures of heading no. 4001 and 4002, in primary forms or in plates, sheets or strip
4003	Reclaimed rubber in primary forms or in plates, sheets or strip
4004	Waste, parings and scrap of rubber (other than hard rubber) and powders and granules obtained therefrom
4005	Compounded rubber, unvulcanised, in primary forms or in plates, sheets or strip
4006	Unvulcanised rubber in other forms (eg rods, tubes and profile shapes) and articles (eg discs and rings)
4007	Vulcanised rubber thread and cord
4008	Plates, sheets, strip, rods and profile shapes, of vulcanised rubber other than hard rubber
4009	Tubes, pipes and hoses, of vulcanised rubber (other than hard rubber), with or without their fittings (eg joints, elbows, flanges)
4010	Conveyor or transmission belts or belting, of vulcanised rubber
4011	New pneumatic tyres, of rubber
4012	Retreaded or used pneumatic tyres of rubber; solid or cushion tyres, interchangeable tyre treads and tyre flaps, of rubber
4013	Inner tubes, of rubber
4014	Hygienic or pharmaceutical articles (including teats), of vulcanised rubber other than hard rubber, with or without fittings of hard rubber
4015	Articles of apparel and clothing accessories (including gloves), for all purposes, of vulcanised rubber other than hard rubber
4016	Articles of vulcanised rubber other than hard rubber, n.e.s. in chapter 40
4017	Hard rubber (eg ebonite) in all forms, including waste and scrap; articles of hard rubber
8701	Tractors; (other than tractors of heading no 8709)
8702	Vehicles; public transport passenger type
8703	Motor cars and other motor vehicles; principally designed for the transport of persons (other than those of heading no. 8702), including station wagons and racing cars
8704	Vehicles; for the transport of goods
8705	Special purpose motor vehicles; not those for the transport of persons or goods (eg breakdown lorries, road sweeper lorries, spraying lorries, mobile workshops, mobile radiological units etc)
8711	Motorcycles (including mopeds) and cycles; fitted with an auxiliary motor, with or without side-cars; side-cars
8712	Bicycles and other cycles; including delivery tricycles, not motorised
8716	Trailers and semi-trailers; other vehicles, not mechanically propelled; parts thereof

Table S4: Natural, synthetic, and reclaimed rubber content of traded rubber goods. For each category (e.g. 4001), we selected one item to exemplify, based on the information we had available. These items might not be fully representative of all items included in each category.

Code	Description	Example item	Rubber content (% of item weight)	New natural rubber content (4001) (%)	New synthetic rubber content (4002) (%)	Reclaimed rubber (4003) (%)	Conversion factor
4001	Natural rubber, balata, gutta-percha, guayule, chicle and similar gums; in primary forms or in plates, sheets or strip		100	100	0	0	1
4002	Synthetic rubber and factice derived from oils, in primary forms or in plates, sheets or strip; mixtures of heading no. 4001 and 4002, in primary forms or in plates, sheets or strip		0	0	100	0	0
4003	Reclaimed rubber in primary forms or in plates, sheets or strip	Note on reclaimed rubber [1]	50 to 60	0	0	100	0
4004	Waste, parings and scrap of rubber (other than hard rubber) and powders and granules obtained therefrom	Note on reclaimed rubber [1]	50 to 60	0	0	100	0
4005	Compounded rubber, unvulcanised, in primary forms or in plates, sheets or strip	Natural Rubber (NR) Study Formulations—Factory Trials [1]	66	100	0	0	0.66
4006	Unvulcanised rubber in other forms (e.g. rods, tubes and profile shapes) and articles (e.g. discs and rings)	Tire Tread or Camel Back 1 [1]	61	100	0	0	0.61
4007	Vulcanised rubber thread and cord	Cord Friction Compound [1]	77	100	0	0	0.77
4008	Plates, sheets, strip, rods and profile shapes, of vulcanised rubber other than hard rubber	Rubber Strap for Microcellular/Sponge Soling [1]	61	100	0	0	0.61
4009	Tubes, pipes and hoses, of vulcanised rubber (other than hard rubber), with or without their fittings (eg joints, elbows, flanges)	Nitrile Rubber Hose Outer [1]	38	0	100	0	0
4010	Conveyor or transmission belts or belting, of vulcanised rubber	Conveyor Belt Cover (Natural Rubber/SBR Blend) [1]	63	25	75	0	0.16
4011	New pneumatic tyres, of rubber (other)	Passenger/ Light truck tires [2]	43	19	81	0	0.08
4012	Retreaded or used pneumatic tyres of rubber; solid or cushion tyres, interchangeable tyre treads and tyre flaps, of rubber	Passenger/ Light truck tires [2]	43	19	81	0	0.08
4013	Inner tubes, of rubber	Automotive Tire Tubes—45 A (share NR and SR by half) [1]	65	50	50	0	0.33
4014	Hygienic or pharmaceutical articles (including teats), of vulcanised rubber other than hard rubber, with or without fittings of hard rubber	Pharmaceutical Bottle Closures [1]	49	0	100	0	0
4015	Articles of apparel and clothing accessories (including gloves), for all purposes, of vulcanised rubber other than hard rubber	Frictioning Compound [1]	30	100	0	0	0.30
4016	Articles of vulcanised rubber other than hard rubber, n.e.s. in chapter 40	Pencil Eraser—Alternate I [1]	12	100	0	0	0.12
4017	Hard rubber (eg ebonite) in all forms, including waste and scrap; articles of hard rubber	Typical ebonite formulation [1]	37	100	0	0	0.37

[1] Chandrasekaran, 2007
[2] USTMA What is in a tire

Table S5: Land footprint of mobility in the EU28, according to minimum and maximum parameters.

ISO3	Country	EU's natural rubber sourcing	Area harvested (thousand ha) (FAO, for 2016)	EU land footprint (thousand ha)		Share area harvested for EU (%)	
				mid value	min – max	mid value	min – max
TOTAL	Total	100%	11575	594	342 – 1415	5%	3 - 12
BGD	Bangladesh	> 1%	67	1	0.6 - 2	1.6%	0.9 - 3.7
BRA	Brazil	> 1%	147	0.04	0.2 – 1	0.2%	0.1 - 0.7
BRN	Brunei Darussalam	> 1%	4	0.01	0.006 – 0.03	0.3%	0.2 - 0.7
CAF	Central African Republic	> 1%	1	0.1	0.06 – 0.2	7.4%	4.1 - 15.5
CHN	China	11%	724	39	23 – 121	5.3%	3.2 - 16.7
CIV	Côte d'Ivoire	14%	277	60	34 - 139	21.8%	12.4 - 50.1
CMR	Cameroon	1%	60	10	6 - 22	16.3%	9.3 - 37.2
COD	Democratic Republic of the Congo	> 1%	59	1	0.7 - 3	2.0%	1.2 - 4.5
COG	Congo	> 1%	3	0.1	0.08 – 0.3	4.9%	2.8 - 11.4
DOM	Dominican Republic	> 1%	0.5	0.00005	0.00003 – 0.0001	0.1%	0.1 - 0.3
ECU	Ecuador	> 1%	14	0.02	0.01 – 0.05	0.1%	0.1 - 0.3
GAB	Gabon	> 1%	17	2	1 - 4	10.4%	6.0 - 24.3
GHA	Ghana	1%	27	4	2 – 9	14.4%	8.2 - 33.4
GIN	Guinea	> 1%	13	2	1 - 6	18.8%	10.8 - 44.1
GTM	Guatemala	> 1%	100	0.7	0.4 – 2	0.7%	0.4 - 1.7
IDN	Indonesia	26%	3637	192	110 – 442	5.3%	3.0 - 12.2
IND	India	1%	455	4	2 – 9	0.9%	0.5 - 2.0
KHM	Cambodia	1%	16	4	2 – 9	24.9%	14.1 - 57.7
LBR	Liberia	1%	96	12	7 - 27	12.1%	6.9 - 28.4
LKA	Sri Lanka	1%	142	4	2 – 9	2.7%	1.5 - 6.6
MEX	Mexico	> 1%	23	0.2	0.1 – 0.8	0.9%	0.6 - 3.5
MMR	Myanmar	1%	293	7	4 – 16	2.3%	1.3 - 5.5
MYS	Malaysia	6%	1078	65	37 – 151	6.1%	3.4 - 14.0
NGA	Nigeria	1%	369	16	9 – 37	4.3%	2.5 - 10.1
PHL	Philippines	1%	223	4	2 – 9	1.7%	1.0 - 3.9
PNG	Papua New Guinea	> 1%	14	0.7	0.4 - 2	5.5%	3.1 - 12.7
THA	Thailand	26%	3095	136	78 – 320	4.4%	2.5 - 10.4
VNM	Viet Nam	7%	621	30	17 - 72	4.9%	2.8 - 11.6

Table S6: Natural rubber use in car tyres per capita according to min and max parameters.

Countries	Population in 2017	Natural rubber use in car tyres per capita (kg)	min – max (kg)
Austria	8797566	0.72	0.72 - 0.94
Belgium	11375158	0.78	0.39 - 1.01
Bulgaria	7075947	0.47	0.23 - 0.61
Croatia	4124531	0.42	0.21 - 0.55
Cyprus	1179680	0.49	0.24 - 0.63
Czech Republic	10594438	0.49	0.24 - 0.63
Denmark	5764980	0.82	0.41 - 1.07
Estonia	1317384	0.79	0.39 - 1.03
Finland	5508214	1.03	0.51 - 1.34
France	66865144	0.90	0.45 - 1.17
Germany	82657002	1.01	0.50 - 1.31
Greece	10754679	0.66	0.33 - 0.86
Hungary	9787966	0.40	0.20 - 0.52
Ireland	4807388	0.85	0.42 - 1.10
Italy	60536709	0.95	0.48 - 1.24
Latvia	1942248	0.49	0.24 - 0.64
Lithuania	2828403	0.73	0.36 - 0.94
Luxembourg	596336	1.10	0.55 - 1.43
Malta	467999	0.43	0.22 - 0.56
Netherlands	17131296	0.78	0.39 - 1.02
Poland	37974826	0.34	0.17 - 0.44
Portugal	10300300	0.62	0.31 - 0.81
Romania	19587491	0.26	0.13 - 0.33
Slovakia	5439232	0.32	0.16 - 0.41
Slovenia	2066388	0.97	0.49 - 1.26
Spain	46593236	0.54	0.27 - 0.70
Sweden	10057698	0.95	0.48 - 1.24
United Kingdom	66058859	0.86	0.43 - 1.12

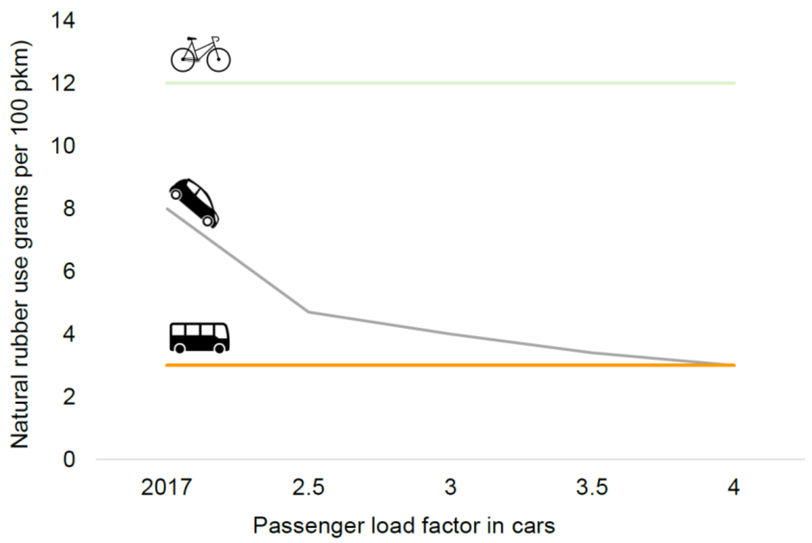

Figure S1: Sensitivity analysis of the "rubber efficiency" of cars compared to bikes and buses. with an increase in the average load factor of cars. Occupancy rates of buses remain at 2017 level.

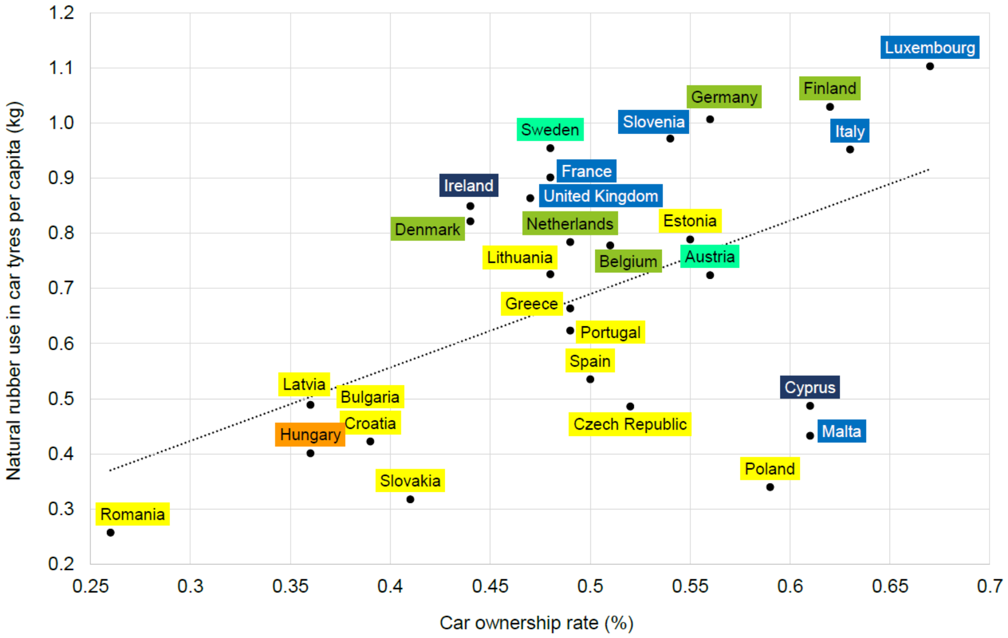

Figure S2: Relationship between car ownership rate and per capita natural rubber use in car tyres in EU 28. Countries are colored according to their classification into "mobility cultures" by Haustein and Nielsen (2016).

In countries towards the bottom right-hand corner of the graph, car ownership does not combine with extensive car use. This may be related to the operating cost of cars which may restrain mobility or encourage ride-sharing, or to the popularity of soft modes such as walking and cycling for everyday travel (Haustein and Nielsen. 2016), which is however shaped by the distance to services (Mattioli et al. 2015). In countries towards the upper left corner of the graph, the distance travelled by car per capita is relatively high despite a moderate level of motorization, certainly in relation to the "car peak" phenomenon described by Focas at al. (2017). This means that cars are used for long-distance travel and that one passenger rides are common in those countries.

Table S7: Projected natural rubber demand and land footprint if all synthetic rubber is replaced by natural rubber in tyres used in the EU.

Country (user)	Initial natural rubber use (tons)	Additional natural rubber use (synthetic) (tons)	Total natural rubber demand (tons)	Land footprint (ha)
Italy	113656	170518	284174	245641
Germany	117542	140237	257779	229529
France	100980	131595	232575	207284
United Kingdom	83925	101669	185594	162418
Spain	42464	68303	110767	96795
Poland	37547	30802	68349	62791
Netherlands	24270	26639	50909	46500
Greece	11117	27901	39018	34899
Belgium	19705	19174	38878	34694
Sweden	14754	16849	31603	27832
Portugal	11882	15415	27297	25261
Austria	10142	12926	23067	20926
Czech Republic	9940	12894	22834	19655
Hungary	8592	10212	18804	16898
Finland	8557	10160	18716	16855
Romania	9641	9193	18835	16259
Denmark	8354	9661	18015	15928
Ireland	7592	9418	17011	14729
Slovakia	5371	7185	12556	11014
Bulgaria	6004	5801	11805	10385
Lithuania	4662	3900	8562	8378
Croatia	3347	5028	8375	7230
Slovenia	3732	3878	7610	6757
Latvia	2464	1899	4363	3838
Luxembourg	1751	2180	3931	3510
Estonia	1646	1675	3321	3189
Cyprus	1029	1567	2596	2155
Malta	414	429	843	735

Table S8: FAOSTAT data sources for natural rubber production, area harvested, and yield. Colors indicate the source. Dark green: official sources, light green: unofficial sources, orange: FAO estimates, FAO data based on imputation methodology, or aggregate (i.e. mix official, semi-official, estimated, and calculated data).

Producer	Production in 2016 (tons)	Proportion of total production in 2016 (%)	Area harvested	Production	Yield (calculated)
Thailand	4519000	31,4%			1
Indonesia	3307142	23,0%			1
Viet Nam	1035333	7,2%			1
India	967175	6,7%			1
China	815938	5,7%			1
China, mainland	815938	5,7%			1
Malaysia	673513	4,7%			1
Côte d'Ivoire	453000	3,2%			1
Philippines	362626	2,5%			1
Guatemala	332755	2,3%			1
Myanmar	221670	1,5%			1
Brazil	189780	1,3%			1
Nigeria	147661	1,0%			1
Cambodia	145200	1,0%			1
Sri Lanka	79100	0,6%			1
Mexico	62351	0,4%			1
Cameroon	50000	0,3%			1
Liberia	47223	0,3%			1
Gabon	25128	0,2%			1
Ghana	22427	0,2%			1
Ecuador	20064	0,1%			1
Bangladesh	18800	0,1%			1
Guinea	16354	0,1%			1
DRC	12000	0,1%			1
Colombia	11800	0,1%			Excluded
Papua New Guinea	7700	0,1%			1
Bolivia	6400	0,0%			Excluded
Congo	2479	0,0%			1
Central African Republic	1150	0,0%			1
Brunei Darussalam	253	0,0%			1
Dominican Republic	60	0,0%			1
Costa Rica	*NA*	*NA*			Excluded
Guinea-Bissau	*NA*	*NA*			Excluded
Peru	*NA*	*NA*			Excluded
Singapore	*NA*	*NA*			Excluded
Timor-Leste	*NA*	*NA*			Excluded
From official sources				53%	
From unofficial sources				33%	

Icons in the manuscript were retrieved from The Noun Project. Use allowed under Creative Commons CCBY Licence. Authors: Yi Chen (car), Pierre-Luc Auclair (bus), Icolabs (motorcycle), Malena Zook (bike), Nikita Kozon (metro), Lee Mette (train, van), Jon Trillana (truck).
